# Four New Cyclohexylideneacetonitrile Derivatives from the Hypocotyl of Mangrove (*Bruguiera gymnorrhiza*)

**DOI:** 10.3390/molecules200814565

**Published:** 2015-08-12

**Authors:** Xiang-Xi Yi, Jia-Gang Deng, Cheng-Hai Gao, Xiao-Tao Hou, Fei Li, Zhi-Ping Wang, Er-Wei Hao, Yan Xie, Zheng-Cai Du, Hui-Xue Huang, Ri-Ming Huang

**Affiliations:** 1 School of Pharmaceutical Sciences, Guangxi University of Chinese Medicine, Nanning 530001, China; E-Mails: xiangxiyi81@aliyun.com (X.-X.Y.); dengjg53@126.com (J.-G.D.); xthou@126.com (X.-T.H.); wangzhiping@163.com (Z.-P.W.); ewhao@163.com (E.-W.H.); xiey2009@126.com (Y.X.); duzhengcai8@163.com (Z.-C.D.); hhx123@hotmail.com (H.-X.H.); 2 Guangxi Key Laboratory of Marine Environmental Science, Guangxi Academy of Sciences, Nanning 530007, China; E-Mails: gaochenghai@gxas.cn (C.-H.G.); finylee@yeah.net (F.L.); 3 Key Laboratory of Plant Resources Conservation and Sustainable Utilization, South China Botanical Garden, Chinese Academy of Sciences, Guangzhou 510650, China; 4 Guangdong Provincial Key Laboratory of Applied Botany, South China Botanical Garden, Chinese Academy of Sciences, Guangzhou 510650, China

**Keywords:** antiviral, *Bruguiera gymnorrhiza*, cyclohexylideneacetonitrile derivative

## Abstract

Four new cyclohexylideneacetonitrile derivatives **1**–**4**, named menisdaurins B–E, as well as three known cyclohexylideneacetonitrile derivatives—menisdaurin (**5**), coclauril (**6**), and menisdaurilide (**7**)—were isolated from the hypocotyl of a mangrove (*Bruguiera gymnorrhiza*). The structures of the isolates were elucidated on the basis of extensive spectroscopic analysis. Compounds **1**–**7** showed anti-Hepatitis B virus (HBV) activities, with EC_50_ values ranging from 5.1 ± 0.2 μg/mL to 87.7 ± 5.8 μg/mL.

## 1. Introduction

**Figure 1:**
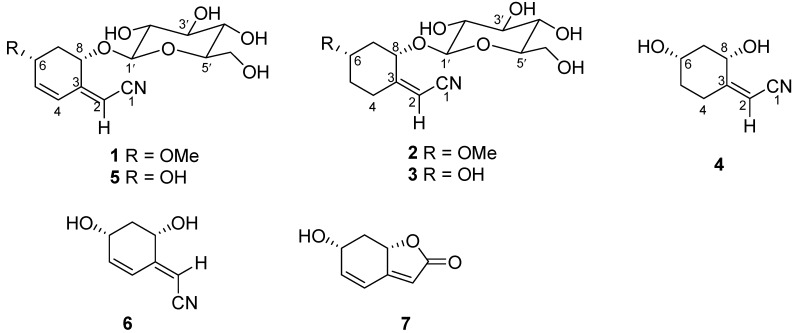
Secondary metabolites **1**–**7**.

*Bruguiera gymnorrhiza* (L.) Savigny (Rhizophoraceae) is a common buttressed tree found in the mangrove forests [1] which are native to many countries of southern and eastern Africa, Asia, and northern Australia [2]. Different parts of these mangrove plants have traditionally been used as herbal medicines in Thailand and China [2]. Previous phytochemical investigations on *B. gymnorrhiza* have shown the presence of diterpenes, triterpenes, flavonoids, aromatic compounds, and sulfur-containing compounds [1,2,3,4,5,6]. As part of our continuing program aimed at exploring the bioactive natural products from mangrove plants collected on the coast of South China Sea [7,8], four new cyclohexylideneacetonitrile derivatives **1**–**4** (Figure 1), which have been named menisdaurins B–E, as well as three known cyclohexylideneacetonitrile derivatives—menisdaurin (**5**) [9], coclauril (**6**) [10], and menisdaurilide (**7**) [11]—were isolated from the hypocotyl of a mangrove *B. gymnorrhiza*. Herein, we will discuss the isolation, structural elucidation, and antiviral activity of these secondary metabolites.

## 2. Results and Discussion

The molecular formula of menisdaurin B (**1**), a yellow powder, was established as C_15_H_21_NO_7_ based on the NMR and HRESIMS data ([M − H]^−^, *m*/*z*: 326.1238; calcd. for C_15_H_20_NO_7_ *m*/*z*: 326.1240). A characteristic, sharp band at 2225 cm^−1^ in the IR spectrum and a signal at δ_C_ 117.9 (C-1) in the ^13^C-NMR showed the presence of an α,β-unsaturated nitrile [9]. The ^1^H- and ^13^C-NMR (Table 1) data suggested the presence of a sugar joined to an aglycone by an α,β-glycosidic linkage (anomeric proton and carbon; δ_H_ 4.38 (1H, d, *J* = 7.8 Hz, H-1′) and δ_C_ 102.3 (C-1′), respectively). The acid hydrolysis of **1** with HCl gave a d-glucose, which was confirmed by TLC. In the ^1^H-NMR spectrum of the aglycone in **1** (Table 1), two olefinic protons at δ_H_ 6.20 (1H, d, *J* = 10.0 Hz, H-4) and 6.18 (1H, dd, *J* = 10.0, 2.5 Hz, H-5), two methine protons at δ_H_ 4.72 (1H, dd, *J* = 10.2, 3.5 Hz, H-8) and 4.45 (1H, ddd, *J* = 9.0, 5.5 and 2.5 Hz, H-6), and one methene proton at δ_H_ 2.39 (1H, ddd, *J* = 12.5, 5.5 and 3.5 Hz, H-7a) and 1.62 (1H, ddd, *J* = 12.5, 10.2 and 9.0 Hz, H-7b), and also from the COSY and HMQC, the sequence from C-4 to C-8 was established as –CH–CH–CH(O)–CH_2_–CH(O)–. The remaining methine signal (δ_C_ 97.2 (C-2) and δ_H_ 5.66 (1H, d, *J* = 1.8 Hz, H-1)) and a quaternary carbon (δ_C_ 155.2 (C-3)) resembled the cyanomethylene group of menisdaurin (**1**) [9] also isolated from the same plant *B. gymnorrhiza*, whose structure was confirmed by comparison the [α]_D_, IR spectrum and detailed NMR data with those in the literature [9,12,13]. In contrast to the low optical rotation of a ${\left[\alpha \right]}_{\text{D}}^{22}$ (−145°, in MeOH, *c* 0.5) reported for menisdaurin in [12], we measured an ${\left[\alpha \right]}_{\text{D}}^{20}$ (−78.2°, in MeOH, *c* 0.2) for our isolated menisdaurin (**5**). Although this differs from the ${\left[\alpha \right]}_{\text{D}}^{22}$ value given for menisdaurin in [12], it is acceptable considering the compound **5** was the same substance, namely menisdaurin. This is underlined by a characteristic sharp band at 2223 cm^−^^1^ in the IR spectrum and a signal at δ_C_ 118.5 (C-1) in the ^13^C-NMR showed the presence of an α,β-unsaturated nitrile, which are in good agreement with those of menisdaurin in [9,13]. Finally the detailed NMR data given for **5** fit the reported data of menisdaurin in [9,12,13].

**Figure 2:**
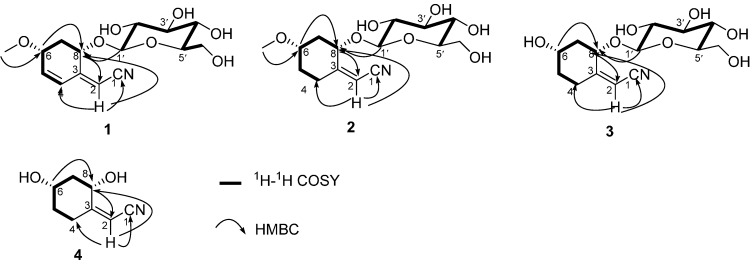
Selected ^1^H-^1^H COSY and HMBC correlations of **1**–**4**.

The spin systems of H-1′/H-2′/H-3′/H-4′/H-5′/H_2_-6′ and H-4/H-5/H-6/H_2_-7/H-8 present in **1**, as the analysis of the ^1^H-^1^H COSY correlations revealed, were assembled with the assistance of the HMBC correlations (Figure 2). The structure of **1** was further confirmed by the HMBC spectrum (Figure 2). There were significant long range couplings between H-2 and C-1, C-4 and C-8, which further established the position of the α,β-unsaturated nitrile. The anomeric proton H-1′ has a long range correlation with C-8 which showed that the sugar moiety is attached to C-8 of the aglycone. Moreover, the methoxyl attached to C-6 was secured by the HMBC correlation of OCH_3_ to C-6.

**Figure 3:**
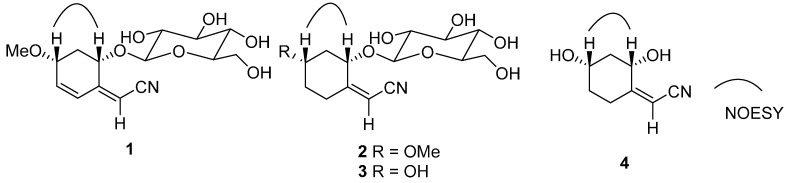
Selected NOESY correlations of **1**–**4**.

The stereochemistry of **1** was established by a comprehensive analysis of the ^1^H-NMR coupling constants, NOESY (Figure 3), ^13^C-NMR and CD data. The large coupling characteristics showed the pseudodiaxial relationships of H-7b (ddd, *J*_7b,8_ = 10.2 Hz, *J*_7b,6_ = 9.0 Hz, and *J*_7a,7b_ = 12.5 Hz) with both H-6 and H-8, together with a NOE correlation between H-6 and H-8 in the NOESY spectrum (Figure 3), and demonstrated that H-6, H-7b and H-8 occupied the pseudodiaxial positions and that H-6 and H-8 were oriented on the same side of the ring system in **1**. Compound **1** showed a ${\left[\alpha \right]}_{\text{D}}^{20}$ in MeOH of −44.7°. The reported value for menisdaurin was negative (${\left[\alpha \right]}_{\text{D}}^{22}$ −145°) [12], whose stereochemistry of the aglycone and β-glucosyl residue have been established by the X-ray single crystallographic analysis [14] and enzymatic hydrolysis [15], respectively. Moreover, the NMR data of in C-8 (δ_C_ 73.2) in **1** is identical to that of C-8 (δ_C_ 73.2) observed in menisdaurin [13], which indicated that C-8 in **1** has the same absolute stereostructure as C-8 in menisdaurin. Finally, the absolute configurations of C-6 and C-8 were further confirmed by its CD spectrum, in which a negative Cotton effect by the cyclohexene group was show at 215 nm (Δε_215nm_−25.5). The appplicaton of the octant rule [16] to the compound depicted in the formula of **1** found that the expected sign of the Cotton effect should be negative. Accordingly, the absolute configurations at the chiral centres C-6 and C-8 of the aglycone in **1** were assigned as *S* and *R*, respectively. The structure of **1** is thus predicted to be as shown in Figure 1.

Menisdaurin C (**2**), was obtained as a yellow powder, and had the molecular formula C_15_H_23_NO_7_ as deduced from the HR-ESI-MS ([M − H]^−^, *m*/*z*: 328.1394; calcd for C_15_H_22_NO_7_ *m*/*z*: 328.1396) and NMR data (Table 1). Furthermore, its NMR spectra and the sharp band at 2222 cm^−1^ in the IR spectrum established that **1** possessed an α,β-unsaturated nitrile [9] and a β-glucosyl moiety, which were greatly similar to those of **1** and menisdaurin (**5**), indicating that **2**, **1**, and **5** are structurally related. A careful comparison of the ^1^H- and ^13^C-NMR spectra of **2** with those of **1** revealed that the C-4 and C-5 are double bonds in **1**, while C-4 and C-5 in **2** are methylene groups.

It was possible to differentiate between the separate spin systems of H-1′/H-2′/H-3′/H-4′/H-5′/H_2_-6′ and H_2_-4/H_2_-5/H-6/H_2_-7/H-8 from the ^1^H-^1^H COSY spectrum of **2** (Figure 2). These data, together with the key HMBC correlations between H-1′/C-8; H-2/C-1, C-4, and C-8; and MeO-6/C-6 (Figure 2), permitted the elucidation of the carbon skeleton of **2**.

The relative configuration of the aglycone in **2** was determined by the NOESY experiment (Figure 3) and ^1^H-NMR *J* values. In the ^1^H-NMR, a large coupling constant (*J*_7b,8_ = 9.7 Hz) was observed for H-8, which required a *trans*-diaxial relationship between H-8 and H-7b. In addition, a NOESY correlation between H-8 and H-6 indicated that these two protons were in the axial orientations and *cis* to one another.

Compound **2** showed a ${\left[\alpha \right]}_{\text{D}}^{20}$ in MeOH of −56.2°. The corresponding reported value for menisdaurin was negative (${\left[\alpha \right]}_{\text{D}}^{22}$ −145°) [12], and the observed value for **1** was −44.7°. The NMR data of C-8 (δ_C_ 73.9) in **2** was very similar to that observed at C-8 (δ_C_ 73.2) in compound **1** and C-8 (δ_C_ 73.2) in menisdaurin [13], which suggested that the absolute configuration of C-8 was the same as those of C-8 in compound **1** and menisdaurin. Furthermore, the absolute configurations of C-6 and C-8 were confirmed by its CD spectrum, in which a negative Cotton effect caused by the nitrylidenecyclohexane group appeared at 215 nm (Δε_2__15nm_−22.7), the applicaton of the octant rule to the compound depicted as compound **2** found that the expected sign of the Cotton effect should be negative [17]. Therefore, the absolute configuration of the aglycone in **2** was assigned as *S* and *R*, respectively. Compound **2** is thus determined as shown in Figure 1 on the basis of the above spectroscopic evidence.

Menisdaurin D (**3**), was obtained as a yellow powder, and the molecular formula C_14_H_21_NO_7_ was obtained from the HR-ESI-MS ([M − H]^−^, *m*/*z*: 314.1238; calcd for C_14_H_20_NO_7_ *m*/*z*: 314.1240) and NMR spectral data (Table 1). The molecular formula indicated that **3** was a derivative of **2**. Comparison of the ^1^H- and ^13^C-NMR spectra of **3** with those of **2** confirmed the overall similarity between their structures. However, a careful comparison of the ^1^H- and ^13^C-NMR spectra of **3** with those of **2** revealed that the major differences between **2** and **3** were the 6-OMe in **2** and an especially downfield signal of the hydroxyl group 6-OH at δ_H_ 4.79 in **3**, and the relative up shift of C-6 at δ_C_ 67.2 in **3**.

The molecular framework was established by the ^1^H-^1^H COSY and HMBC correlations (Figure 2). The comprehensive analysis of the ^1^H-^1^H COSY correlations of **3** established the spin systems of H-1′/H-2′/H-3′/H-4′/H-5′/H_2_-6′ and H_2_-4/H_2_-5/H-6/H_2_-7/H-8. The MeO-6 group attached to C-6 was confirmed by the HMBC correlations from MeO-6 to C-6. The planar structure of **3** was further confirmed by the HMBC correlations between H-2 and C-1, C-4, and C-8, which established the position of the α,β-unsaturated nitrile, and the correlations between the anomeric proton H-1′ and C-8, which showed that the sugar moiety is attached to C-8 of the aglycone.

The NOESY correlation of H-8 and H-6 (Figure 3) and the a large coupling constant (*J*_7b,8_ = 9.4 Hz) confirmed that these two protons were cofacial and were at the axial positions. Compound **3** showed a ${\left[\alpha \right]}_{\text{D}}^{20}$ in MeOH of −76.2°. The reported value for menisdaurin was negative (${\left[\alpha \right]}_{\text{D}}^{22}$ −145°) [12], and the observed values for **1** and **2** were −44.7° and −56.2°, respectively. Combined with the NMR data of C-8 (δ_C_ 74.8) in **3** is greatly similar with that of C-8 (δ_C_ 73.9) observed in **2** and the key NOESY correlation of H-6 and H-8, the configurations of C-6 and C-8 in compound **3** should be the same as those of C-6 and C-8 in **2**, namely *S* and *R*, respectively. Moreover, the Cotton effect values of both **2** and **3** showed negative Cotton effects at 215 nm (Δε_2__15nm_−22.7) and (Δε_2__15nm_−42.6), respectively. Compound **3** is identified as a derivative of **2** on the basis of the above spectroscopic evidence.

Menisdaurin E (**4**) was obtained as colorless plates, and the molecular formular C_8_H_11_NO_2_ was obtained from the HR-ESI-MS data ([M + Na]^+^, *m*/*z*: 176.0679; calcd for C_8_H_11_NO_2_Na *m*/*z*: 176.0687) and NMR spectral data (Table 1). A characteristic, sharp band at 2219 cm^−^^1^ in the IR spectrum and a signal at δ_C_ 116.8 (C-1) in the ^13^C-NMR showed the presence of an α,β-unsaturated nitrile [9]. Analyses of the ^13^C-NMR data indicated that **4** is the aglycone of compound **3**.

The molecular framework was established by the ^1^H-^1^H COSY and HMBC correlations (Figure 2). The comprehensive analysis of the ^1^H-^1^H COSY correlations of **4** established the spin system of H_2_-4/H_2_-5/H-6/H_2_-7/H-8. The planar structure of **4** was further confirmed by the HMBC correlations between H-2 and C-1, C-4, and C-8, which established the position of the α,β-unsaturated nitrile.

The NOESY correlation of H-8 and H-6 (Figure 3) and the large coupling constant (*J*_7b,8_ = 9.3 Hz) confirmed that these two protons were cofacial and were at the axial positions. Compound **4** showed a ${\left[\alpha \right]}_{\text{D}}^{20}$ in MeOH of −47.3°. The observed value for **3** was −76.2°. Moreover, the Cotton effect values of both **3** and **4** showed negative Cotton effects at 215 nm (Δε_2__15nm_ −42.6) and (Δε_2__15nm_ −33.4), respectively. Compound **4** is identified as the aglycone of **3** on the basis of the above spectroscopic evidence.

A comparison of the anti-HBV activities for selected sets of **1**–**7** are displayed in Table 2. The standard deviations listed in Table 2 for the EC_50_ and CC_50_ values were calculated using the coefficients of variance produced by each regression analysis. A selectivity index (SI) was calculated for each compound and is expressed as the ratio of CC_50_ to EC_50_ (Table 2).

**Table 1:** ^1^H- and ^13^C-NMR data of **1**–**4**.

No.	δ_C_, Mult	1 ^a^		2 ^a^		3 ^a^		4 ^b^
		δ_H_ (*J* in Hz)	δ_C_, Mult	δ_H_ (*J* in Hz)	δ_C_, Mult	δ_H_ (*J* in Hz)	δ_C_, Mult	δ_H_ (*J* in Hz)
1	117.9, C		117.1, C		118.7, C		116.8, C	
2	97.2, CH	5.66 (d, 1.8)	94.7, CH	5.53 (d, 1.5)	94.3, CH	5.51 (d, 1.8)	89.4, CH	5.6 (s)
3	152.2, C		166.0, C		166.5, C		170.5, C	
4a	125.4, CH	6.20 (d, 10.0)	29.7, CH_2_	2.46 (d, 11.9)	29.6, CH_2_	2.45 (d, 11.0)	25.2, CH_2_	2.79 (d, 11.0)
4b				2.25 (dd, 11.9, 7.8)		2.11 (dd, 11.0, 7.8)		2.59 (dd, 11.0, 7.8)
5a	144.3, CH	6.18 (dd, 10, 2.5)	36.4, CH_2_	1.86–1.92 (m)	36.3, CH_2_	1.69–1.74 (m)	33.5, CH_2_	1.87–1.93 (m)
5b				1.19–1.24 (m)		1.36–1.42 (m)		1.61–1.67 (m)
6	62.8, CH	4.45 (ddd, 9.0, 5.5, 2.5)	63.9, CH	3.92–3.87 (m)	67.2, CH	3.75–3.70 (m)	67.7, CH	4.48–4.54 (m)
7a	37.1, CH_2_	2.39 (ddd, 12.5, 5.5, 3.5)	36.1, CH_2_	1.87 (ddd, 11.5, 5.2, 3.5)	41.1, CH_2_	2.09 (ddd, 11.3, 5.0, 3.8)	42.9, CH_2_	2.18 (ddd, 10.1, 5.0, 3.3)
7b		1.62 (ddd, 12.5, 10.2, 9.0)		1.17 (ddd, 11.5, 9.7, 8.8)		1.55 (ddd, 11.3, 9.4, 8.5)		1.56 (ddd, 10.1, 9.3, 8.5)
8	73.2, CH	4.72 (dd, 10.2, 3.5)	73.9, CH	3.08 (dd, 9.7, 3.5)	74.8, CH	3.10 (dd, 9.4, 3.8)	65.7, CH	4.17 (dd, 9.3, 3.3)
1′	102.3, CH	4.38 (d, 7.8)	102.8, CH	4.15 (d, 7.3)	102.6, CH	4.27 (d, 7.6)		
2′	73.8, CH	3.16 (ddd, 8.7, 7.8, 4.5)	75.9, CH	2.95 (ddd, 8.5, 7.3, 4.3)	75.8, CH	3.09 (ddd, 8.4, 7.6, 4.3)		
3′	77.4, CH	3.39 (ddd, 8.7, 8.7 4.2)	77.6, CH	3.10 (ddd, 8.7, 8.5, 4.5)	78.8, CH	3.11 (ddd, 8.4, 8.3, 4.5)		
4′	70.6, CH	3.01 (ddd, 8.7, 8.7, 5.4)	70.4, CH	3.04 (ddd, 8.7, 8.7, 5.2)	71.9, CH	3.01 (ddd, 8.3, 7.8, 5.2)		
5′	77.3, CH	3.11 (ddd, 8.7, 6.8, 6.2)	77.5, CH	2.94 (ddd, 8.7, 6.4, 2.4)	78.6, CH	3.05 (ddd, 7.8, 6.4, 2.2)		
6′a	61.8, CH_2_	3.45 (ddd, 11.8, 6.2, 5.2)	61.4, CH_2_	3.39 (ddd, 11.3, 6.2, 5.1)	63.0, CH_2_	3.39 (ddd, 11.0, 6.2, 5.1)		
6′b		3.98 (ddd, 11.8, 6.8, 2.3)		3.65 (ddd, 11.3, 6.8, 2.2)		3.65 (ddd, 11.0, 6.5, 2.2)		
6-OMe	49.2, CH_3_		49.2, CH_3_					
6-OH						4.79 (d, 3.5)		
2′-OH		4.97 (d, 4.5)		5.09 (d, 4.3)		5.02 (d, 4.3)		
3′-OH		4.94 (d, 4.2)		5.04 (d, 4.5)		5.01 (d, 4.5)		
4′-OH		4.93 (d, 5.4)		5.02 (d, 5.2)		4.99 (d, 5.2)		
6′-OH		4.17 (d, 5.2)		4.15 (d, 5.1)		4.13 (d, 5.1)		

^a^ In DMSO-*d*_6_, 600 MHz for ^1^H- and 150 MHz for ^13^C-NMR; ^b^ In CD_3_OD, 600 MHz for ^1^H- and 150 MHz for ^13^C-NMR.

**Table 2:** Anti-HBV activity of **1**–**7**.

Compounds	EC_50_ (μg/mL)	CC_50_ (μg/mL)	SI (CC_50_/EC_50_)
**1**	23.8 ± 0.5	178.2 ± 13.3	7.5
**2**	30.7 ± 1.0	164.9 ± 10.4	5.4
**3**	19.4 ± 0.3	182.0 ± 6.8	9.4
**4**	8.7 ± 1.1	72.3 ± 4.1	8.3
**5**	5.1 ± 0.2	57.8 ± 3.9	11.3
**6**	7.6 ± 0.5	83.9 ± 2.3	11.0
**7**	80.7 ± 5.8	214.8 ± 14.7	2.7
**Lamivudine**	0.1 ± 0.03	/	/

According to the results described in Table 2, it is easy to understand that most of the tested compounds **1**–**7** showed moderate antiviral properties. In general, this study revealed the activity order as **5** > **6** > **4** > **3** > **1** > **2** > **7**. Compound **5** had the most effective activity against HBV replication in the human hepatoblastoma cell line. Compound **7**, by comparison, demonstrated a lesser degree of specific activity against HBV replication.

## 3. Experimental Section

### 3.1. General Procedures

UV spectra were recorded in MeOH on a Lambda 35 UV-Vis spectrophotometer (Perkin-Elmer, Wellesley, MA, USA). The IR spectra were measured in KBr on a WQF-410 FT-IR spectrophotometer (Beifen-Ruili, Beijing, China). NMR spectra were recorded on an AV 600 MHz NMR spectrometer with TMS as an internal standard (Bruker, Bremen, Germany). HRESIMS data were obtained from a Bruker Maxis mass spectrometer (Bruker). A Waters-2695 HPLC system, using a Sunfire™ C_18_ column (150 mm × 10 mm i.d., 10 μm, Waters, Milford, MA, USA) coupled to a Waters 2998 photodiode array detector was used. Optical rotation data were measured by a Perkin-Elmer Model 341 polarimeter. CD spectra were recorded on a MODEL J-810-150S spectropolarimeter (MODEL J-810-150S, Tokyo, Japan). The silica gel GF_254_ used for TLC was supplied by the Qingdao Marine Chemical Factory (Qingdao, China). Spots were detected on TLC under UV light or by heating after spraying with 5% H_2_SO_4_ in EtOH. All solvent ratios are measured *v*/*v*.

### 3.2. Plant Material

The hypocotyl of *B. gymmorrhiza* was collected from Beilun, Guangxi Province, China, in May 2012. The specimen was identified by Professor Hangqing Fan from the Guangxi Mangrove Research Center, Guangxi Academy of Sciences. A voucher specimen (2012-GXAS-001) was deposited in the Guangxi Key Laboratory of Marine Environmental Science, Guangxi Academy of Sciences, China.

### 3.3. Extraction and Isolation

The hypocotyls of *B. gymmorrhiza* (25.4 kg) were exhaustively extracted with EtOH–CH_2_Cl_2_ (2:1, 30 L) at 25 °C for 4 days three times in a large metal bowl (diameter 80 cm, volume 50 L). The solvent was evaporated *in vacuo* (0.09 MPa, 40 °C, rotary evaporator) to afford a syrupy residue (435 g) that was suspended in distilled water (1.5 L) and extracted successively with petroleum ether (3 × 2 L), ethyl acetate (3 × 2 L) and *n*-butanol (3 × 2 L). The ethyl acetate portion (52.3 g) was subjected to column chromatography on silica gel, using CHCl_3_/Me_2_CO (from 10:0 to 5:4) and CHCl_3_/MeOH (from 10:1 to 0:10) as gradient eluents, giving twelve fractions (A−L). Fraction E was subjected to column chromatography on silica gel, using petroleum/ethyl acetate (from 10:0 to 0:10) as an eluent, giving six sub-fractions (E1−E6), then the sub-fraction E6 was separated by HPLC, using mixtures of MeOH/H_2_O (5:95) to yield **7** (4.1 mg, R_t_ = 27.0 min). Fraction F was separated by HPLC, using mixtures of MeOH/H_2_O (10:90) to yield **6** (1.6 mg, R_t_ = 18.7 min). Fraction G was separated by HPLC, using mixtures of MeOH/H_2_O (5:95) to yield **4** (1.2 mg, R_t_ = 19.0 min). The *n*-butanol soluble portion (171.4 g) was subjected to column chromatography on silica gel, using CHCl_3_/MeOH (from 10:0 to 0:10) as an eluent, giving fourteen fractions (A_n_−N_n_). Fraction N_n_ was subjected to a Sephadex LH-20 column chromatography with MeOH, then separated by HPLC, using mixtures of MeOH/H_2_O (5:95) to yield **5** (1.6 mg, R_t_ = 15.2 min), **1** (3.4 mg, R_t_ = 27.2 min), **2** (1.9 mg, R_t_ = 33.2 min) and **3** (3.7 mg, R_t_ = 48.3 min), respectively.

*Menisdaurin* *B* (**1**): Yellow powder; ${\left[\alpha \right]}_{\text{D}}^{20}$ −44.7° (*c* 0.54, MeOH); CD (MeOH) Δε_2__15nm_−25.5; UV (MeOH) λ_max_ (log ε) nm: 271 (4.12); IR (KBr) ν_max_ 3381 (OH), 2225 (CN), and 1621 (C=C) cm^−^^1^; ^1^H- (DMSO-*d*_6_) and ^13^C-NMR (DMSO-*d*_6_), see Table 1; HRESIMS *m*/*z* 326.1238 (calcd for C_15_H_21_NO_7_ − H, 326.1240).

*Menisdaurin* *C* (**2**): Yellow powder; ${\left[\alpha \right]}_{\text{D}}^{20}$ −56.2° (*c* 0.47, MeOH); CD (MeOH) Δε_2__15nm_−22.7; UV (MeOH) λ_max_ (log ε) nm: 267 (3.72); IR (KBr) ν_max_ 3398 (OH), 2222 (CN), and 1620 (C=C) cm^−^^1^; ^1^H- (DMSO-*d*_6_) and ^13^C-NMR (DMSO-*d*_6_), see Table 1; HRESIMS *m*/*z* 328.1394 (calcd for C_15_H_23_NO_7_ − H, 328.1396).

*Menisdaurin* *D* (**3**): Yellow powder; ${\left[\alpha \right]}_{\text{D}}^{20}$ −76.2° (*c* 0.63, MeOH); CD (MeOH) Δε_2__15nm_−42.6; UV (MeOH) λ_max_ (log ε) nm: 270 (4.53); IR (KBr) ν_max_ 3428 (OH), 2220 (CN), and 1623 (C=C) cm^−^^1^; ^1^H- (DMSO-*d*_6_) and ^13^C-NMR (DMSO-*d*_6_), see Table 1; HRESIMS *m*/*z* 314.1238 (calcd for C_14_H_21_NO_7_ − H, 314.1240).

*Menisdaurin* *E* (**4**): Colorless plates; ${\left[\alpha \right]}_{\text{D}}^{20}$ −47.3° (*c* 0.51, MeOH); CD (MeOH) Δε_2__15nm_−33.4; UV (MeOH) λ_max_ (log *ε*) nm: 269 (4.51); IR (KBr) ν_max_ 3425 (OH) and 2219 (CN) cm^−^^1^; ^1^H (CD_3_OD) and ^13^C-NMR (CD_3_OD), see Table 1; HRESIMS *m*/*z* 176.0679 (calcd for C_8_H_11_NO_2_ + Na, 176.0687).

### 3.4. Acid Hydrolysis of ***1**–**3***

Acid hydrolysis of **1**−**3** afforded glucose, which was identified in comparison with standard sugars as described in the literature [15,18,19]. Compounds **1**−**3** (1 mg each) were hydrolysed with 2 mol/L of HCl in H_2_O for 6 h at 85 °C. The reaction mixtures were concentrated. The reaction mixtures of **1**−**3** and the authentic D-glucose were subjected to TLC, using Me_2_CO/CH_3_CH(OH)CH_3_/H_2_O (26:14:7) as an eluent. The R_f_ values for the reaction mixtures of **1**−**3** (R_f_ for compounds **1**−**3** were 0.382, 0.383, and 0.382, respectively) were greatly similar to this of the authentic D-glucose (R_f_ = 0.384) under the same conditions.

### 3.5. Antiviral Assay

Antiviral activity and toxicity of **1**−**7** were assessed using a standardized culture assay [20], which uses cultures of the HBV-producing, human hepatoblastoma cell line [21]. This cell line, which chronically produces infectious HBV [22], has been shown to be an accurate and predictive model for all measured aspects of cellular HBV replication [23].

## 4. Conclusions

To our best knowledge, cyclohexylideneacetonitrile derivatives have been found in many sources [10,13,24,25,26,27,28], and some of molecules possessed prominent biological properties, such as anti-HBV [14], antioxidant [29], insecticidal [30], antifeedant [30], and antifungal [30] activities. In our work, four new cyclohexylideneacetonitrile derivatives, menisdaurins B–E (compounds **1**–**4**), as well as three known cyclohexylideneacetonitrile derivatives, menisdaurin (**5**), coclauril (**6**), and menisdaurilide (**7**), were isolated from the hypocotyl of the mangrove *B. gymnorrhiza*. Compounds **1**–**7** showed moderate anti-HBV activities. Compounds **1**–**7** were isolated for the first time from *B. gymnorrhiza*, which enriches the class of cyclohexylideneacetonitrile molecules originated from marine resources.
